# Supplementary material for: Interaction with the Assembly Chaperone Ump1 Promotes Incorporation of the β7 Subunit into Half-Proteasome Precursor Complexes Driving Their Dimerization
Source: Biomolecules. 2022 Feb 4;12(2):253. doi: 10.3390/biom12020253 (PMC8961534; doi:10.3390/biom12020253)
Supplement: Supplementary file 1 [file biomolecules-12-00253-s001.zip › biomolecules-1578273-supplementary.pdf]

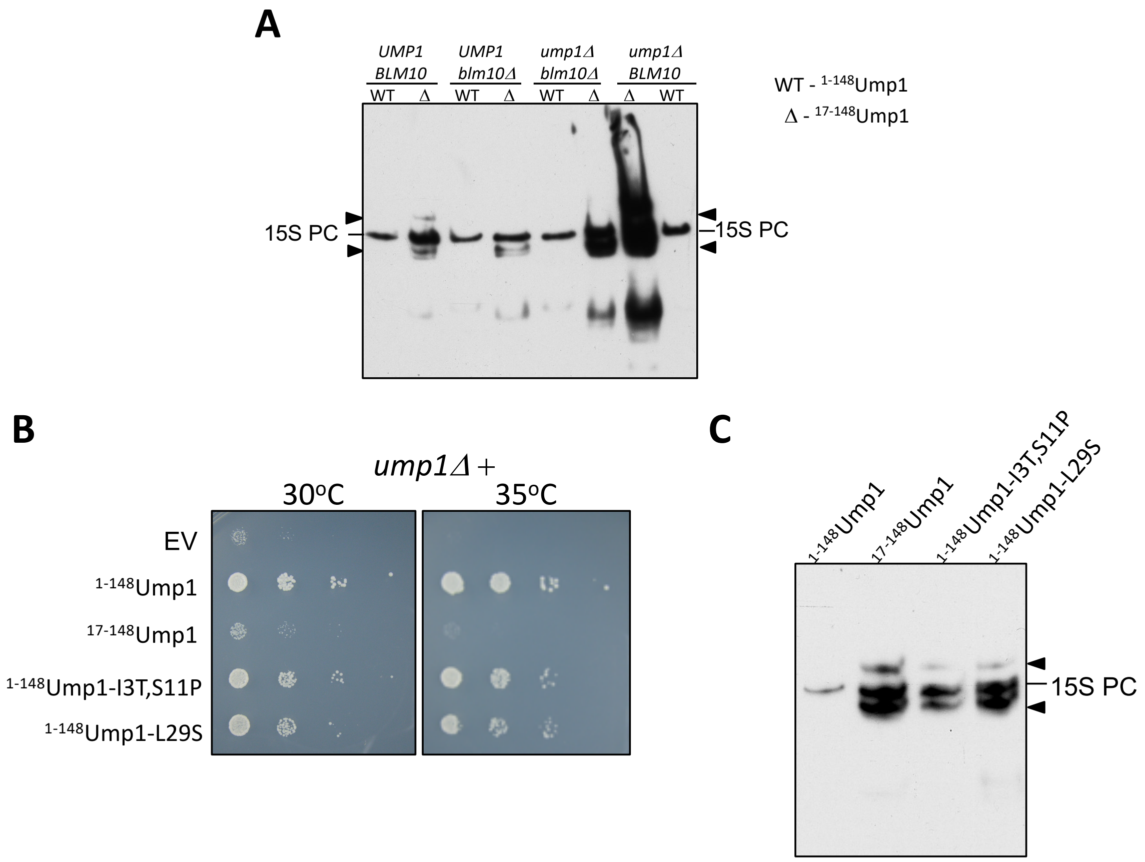

Figure S1. Characterization of complexes in cells with mutant Ump1. (**A**) Native extracts from the indicated strains expressing either Ump1-Ha (WT) or $^{17\text{-}148}$Ump1-Ha (Δ) were analyzed by native PAGE and anti-HA western blotting. (**B**) Spot assays comparing the growth of *ump1*Δ cells expressing the indicated Ump1 versions. Cells were spotted in serial dilutions onto selective SD media and grown for 2 days at the indicated temperatures. (**C**) Analysis of Ump1-HA complexes in the same strains as in (B) by native PAGE and anti-HA western blotting.

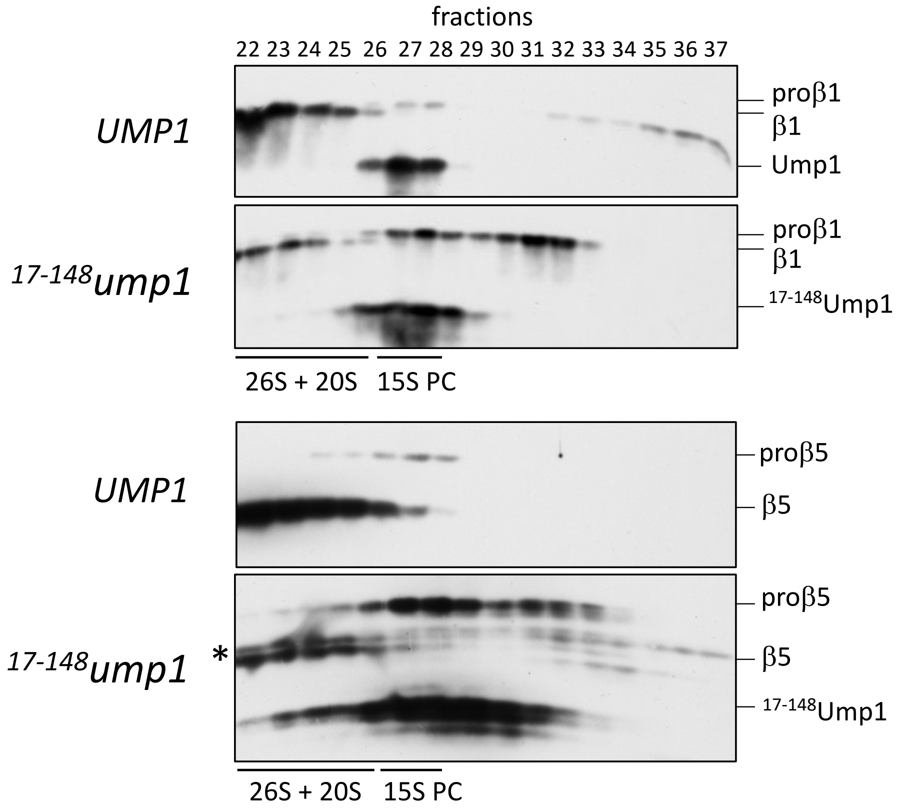

Figure S2. Processing of β5 is incomplete in absence of the first 16 ump1 residues. Crude extracts from cells expressing either β1-HA (top two panels) or β5-HA (bottom two panels) and Ump1-HA were fractionated by Superose 6 gel filtration and analyzed by SDS-PAGE and anti-HA western blotting. The upper panel displays results for the wild-type (*UMP1*), the bottom panel the results for cells with truncated Ump1 ($^{17\text{-}148}$*ump1).* The positions of precursor and mature forms of β subunits and of Ump1 are indicated on the right. An asterisk points to an incompletely processed from of β5 observed in $^{17\text{-}148}$*ump1* cells. Fractions that typically contain the 26S proteasome, the 20S CP, or the 15S PC are indicated at the bottom.

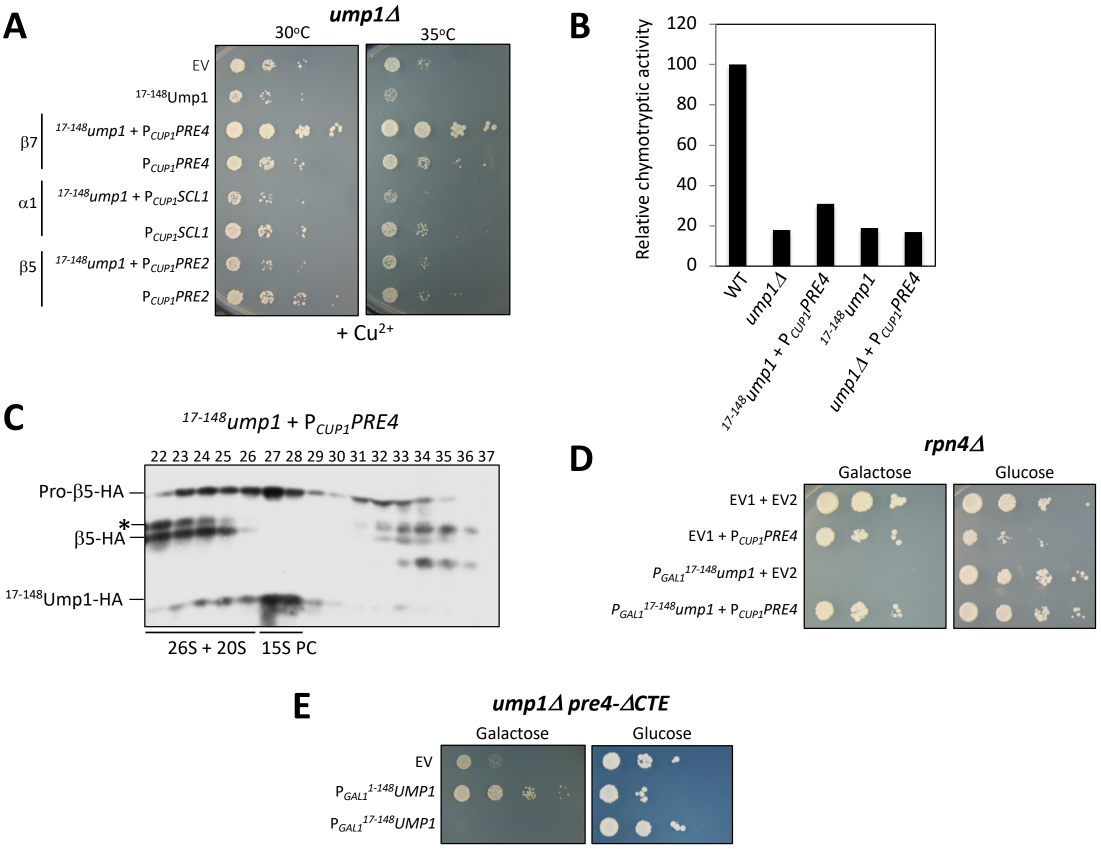

Figure S3. Overexpression of β7 supresses defects caused by $^{17\text{-}148}$Ump1. **(A)** Effect of overexpressing *PRE4*/β7, *PRE2*/β5 and *SCL1*/α1 together on growth of $^{17\text{-}148}$*ump1* cells. EV, empty vector control. **(B)** Effect of β7 overexpression on chymotryptic activity in the indicated backgrounds. **(C)** Effect of β7overexpression on β5 processing analysed by gel filtration. The positions of precursor and mature forms of β subunits and of $^{17\text{-}148}$Ump1 are indicated on the left. An asterisk points to an incompletely processed from of β5 observed in $^{17\text{-}148}$*ump1* cells. **(D)** Overexpression of *PRE4*/β7 suppresses growth defects caused by induction of $^{17\text{-}148}$*ump1* expression from P_{GAL1} in a *rpn4*Δ background. Cells were transformed with two plasmids, either empty vectors (EV1, EV2) and/or with plasmids expressing $^{17\text{-}148}$*ump1* from P_{GAL1} or *PRE4*/β7 from P_{CUP1}. Serial dilutions of the transformed cells were spotted on SD media either with galactose or glucose as carbon sources and incubated at 30°C for 3 or 2 days, respectively. (**E**) Cells lacking the C-terminal extension of Pre4/β7 are hyper-sensitive to $^{17\text{-}148}$*ump1* expression. Cells with the indicated genotype and plasmids were processed as in (D).

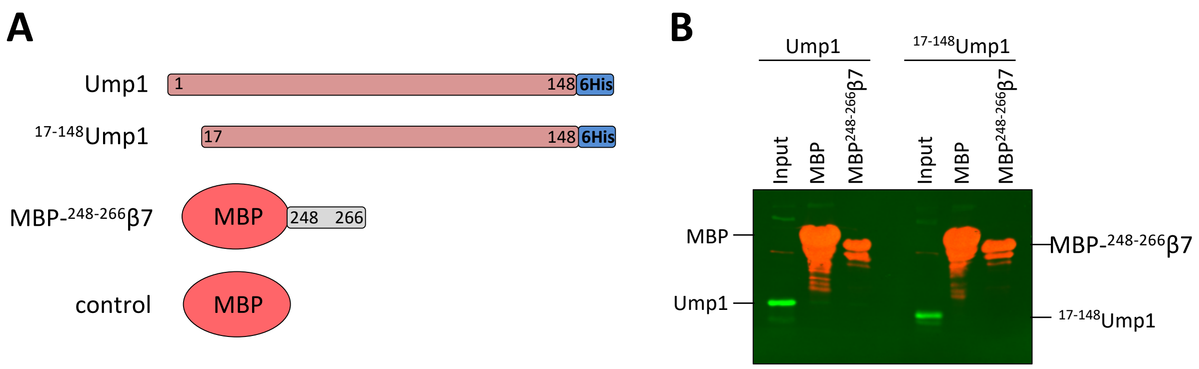

Figure S4. The C-terminal extension of Pre4/β7 by itself does not promote binding to Ump1. **(A)** Schematic representation of 6His tagged Ump1 versions and β7-Cterminus (β7-CT) fused to Maltose Binding Protein (MBP). MBP alone was used as control. **(B)** Western Blot analysis of interactions between Ump1 variants and the C-terminus of β7. Amylose resin was incubated with the native protein extract of 50 OD cells expressing MBP-β7-CT or MBP control. After 5x washing, binding was proceeded with the same amount of Ump1 variants $^{1-148}$Ump1 or $^{16-148}$Ump1. Samples were washed 3x and eluted in 40 µl sample buffer. 10 µl of the samples were loaded on a gel next to 1 % Input of the Ump1 variants and analyzed by Western Blot using anti-β7 and anti-Ump1 antibodies.

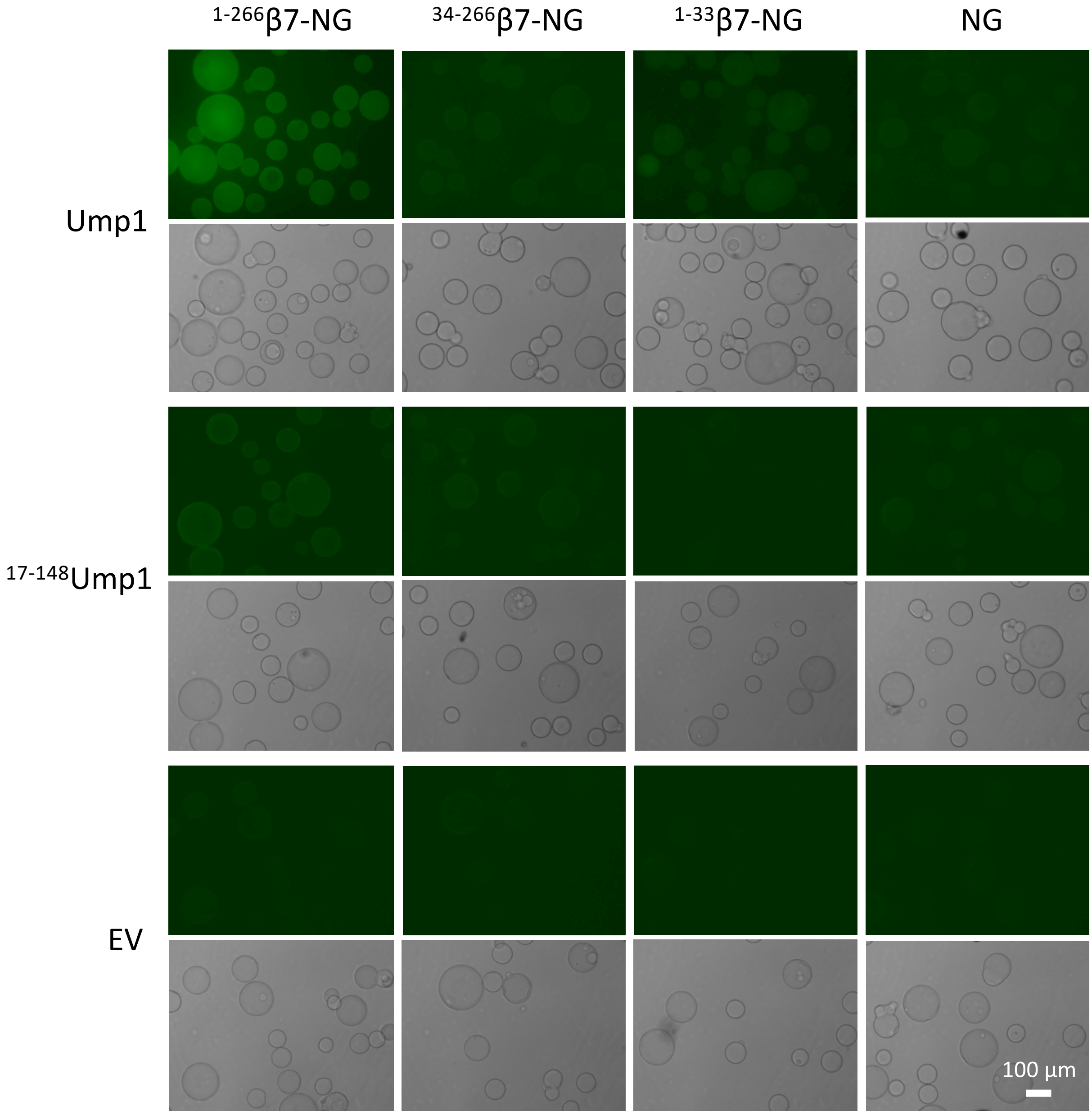

Figure S5. Complete set of pictures including empty vector controls and phase contrast image of beads of experiment shown in figure 5B. The top two rows of green fluorescence are the same as shown in Figure 5B. Shown here, in addition, are images of the empty vector (EV) control that were used as background for the quantitative analysis shown in Figure 5C, and phase contrast images relating to all fluorescence images shown.

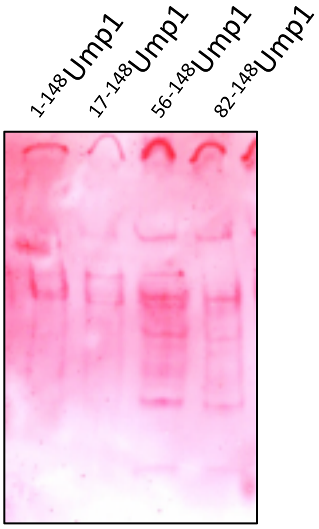

Figure S6. Ponceau S staining of the membrane shown in Figure 1C.

Figure S7. Gel filtration chromatograms (A280/mL) for western blot analyses.

A. Chromatogram for gel filtration shown in Figure 1D

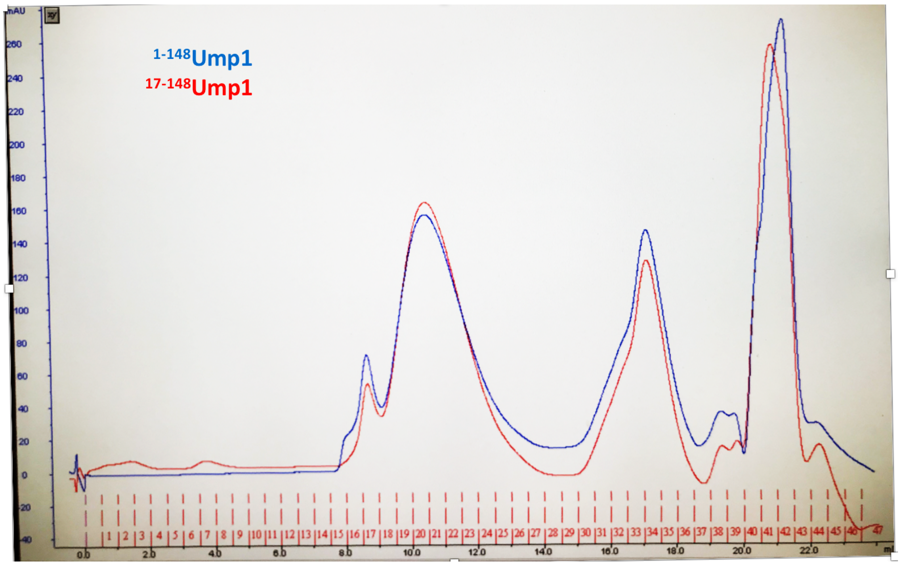

B. Chromatogram for gel filtration shown in Figure 2D

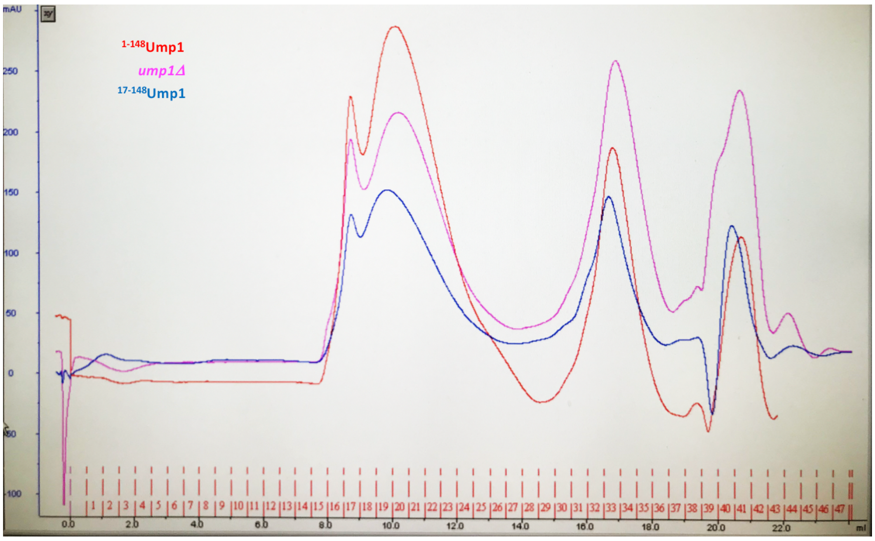

Supplementary Tables

Table S1 – *S. cerevisiae* strains used in this study

Strain*	Relevant Genotype	Source/Derivative
JD47-13C	*MATa his3-Δ200 leu2-3,112 lys2-801 trp1-Δ63 ura3-52*	Dohmen *et al.* 1995 [21]
AM106-2C	*MATa blm10Δ::kanMX6 ump1-Δ::HIS3*	Marques *et al.* 2007 [26]
AM106-1A	*MATa blm10Δ::kanMX6*	Marques *et al.* 2007 [26]
AM144	*MATa pre4-ΔCTE ump1-Δ::HIS3*	Marques *et al.* 2007 [26]
JD59	*MATa ump1Δ::HIS3*	Ramos *et al.* 1998 [5]
ML4	*MATa rpn4Δ::HIS3-MX6*	London *et al.* 2004 [20]
JD134	*MATa ump1Δ::HIS3 PRE2-ha::YIplac211*	Ramos *et al.* 1998 [5]
JD135	*MATa UMP1-2HA::YIplac128 PRE3-2HA::YIplac211*	Ramos *et al.* 1998. [5]
JD136	*MATa ump1Δ::HIS3 PRE3-2HA::YIplac211*	Ramos *et al.* 1998. [5]
JD138	*MATa PRE2-2HA::YIplac211*	Ramos *et al.* 1998. [5]
PR307	*MATa ump1Δ::HIS3 PRE4-2HA::YIplac211*	This work
PR327	*MATa PRE4-HA::YIplac211*	This work

**All strains are congenic derivatives of JD47-13C, which served as a "wild type".*

Table S2 – *S. cerevisiae* plasmids used in this study

Name	Relevant characteristics	Source
pAM5	P_{CUP1}-*PRE4*-T_{CYC1}::YEplac181	Marques *et al.* 2007 [26]
pAM8	P_{CUP1}-*PRE2*-T_{CYC1}::YEplac181	Marques *et al.* 2007 [26]
pAR1	P_{UMP1}-$^{17\text{-}148}$*ump1-HA*::YCplac22	A. Riesener 2002
pAR3	P_{UMP1}-$^{56\text{-}148}$*ump1-HA*::YCplac22	A. Riesener 2002
pAR9	P_{UMP1}-$^{82\text{-}148}$*ump1-HA*::YCplac22	A. Riesener 2002
pAR12	P_{GAL1}-*UMP1-HA*::YEplac181	A. Riesener 2002
pAR4	P_{GAL1}-$^{17\text{-}148}$*ump1-HA*::YEplac181	A. Riesener 2002
pCR42	P_{UMP1}-*ump1-I3T,S11P-HA*::YCplac22	This work
pCR59	P_{CUP1}-*PRE4*-T_{CYC1}::pRS327	This work
pJDNERF22	P_{UMP1}-*UMP1-HA*::YCplac22	This work
pJH57-19	P_{UMP1}-*ump1-L29S-HA*::YCplac22	J. Höckendorff 2000
pHT9	P_{CUP1}-*SCL1*::YEplac181	H. Thran 2004
Rpn4*	*rpn4*$_{\Delta 1\text{-}10/\Delta 211\text{-}229}$::pRS314	Wang *et al.* 2010 [25]
YCplac22	*ARS1-CEN4-TRP1*	Gietz *et al.* 1988 [38]
YEplac181	*2μ-LEU2*	Gietz *et al.* 1988 [38]
pRS314	*ARSH4-CEN6-TRP1*	Sikorski *et al.* 1989 [39]
pRS327	*2μ-LYS2*	Eriksson *et al.* 2004 [40]

Table S3 – *E. coli* plasmids used in this study

Name	Relevant characteristics	Source
pET11a (EV)	P_{T7}; Amp^R	Novagen
pJZ14	8His-*SUMO1-PRE3*	This study
pJZ17	8His-*SUMO1-PRE4*	This study
pJZ18	8His-*SUMO1-ΔLS-pre4* (residues 34-266)	This study
pJZ19	8His-*SUMO1-pre4Δ-CTE* (residues 1-247)	This study
pJZ27	mNG-2HA	This study
pJZ29	*PRE4*-mNG-2HA (β7 precursor form)	This study
pJZ30	$^{1\text{-}33}$*pre4* (β7 pro-peptide, residues 1-33)-mNG-2HA	This study
pJZ33	$^{34\text{-}266}$*pre4* (β7 mature form, residues 34-266)-mNG-2HA	This study
pJZ34	$^{17\text{-}148}$*ump1*-6His	This study
pJD492	*UMP1*-6His	Sá-Moura et al. 2013 [15]
pCR32	$^{1\text{-}81}$*ump1*-6His	This study
pCR33	$^{82\text{-}148}$*ump1*-6His	This study
pCR61	$^{17\text{-}81}$*ump1*-6His	This study
pCR62	$^{1\text{-}81,\ I3T}$*ump*-6His	This study
pCR64	$^{1\text{-}81,\ S11P}$*ump1*-6His	This study
pMAL-c2X	MBP	New England Biolabs
pMO6	MBP-*pre4-CTE* (β7 C-terminal extension, residues 248-266)	M.O. Nunes 2015

Table S4 – Relevant antibodies used in this study

Antibody	Host	Dilution	Source
Anti-HA (3F10)-Peroxidase	rat	1:2000	Roche
Anti-β7 serum, primary	rabbit	1:1000	This study
Anti-β1 serum, primary	rabbit	1:1000	This study
Anti-Ump1, primary	rabbit	1:500	Ramos et al. 1998 [5]
Anti-6His, primary	mouse	1:2000	Invitrogen
Anti-MBP, primary	mouse	1:10000	New England Biolabs
Anti-rabbit Alexa Fluor Plus 800, secondary	goat	1:5000	Thermo-Fisher
anti-mouse Alexa Fluor Plus 680, secondary	goat	1:5000	Thermo Fisher
Anti-Rabbit, HRP	donkey	1:5000	GE Healthcare
Anti-Mouse, HRP	goat	1:5000	Sigma-Aldrich

Supplementary method

Binding assay on amylose resin

50 µl Amylose resin (New England BioLabs) per sample were equilibrated 2x with 1 ml Buffer A (50 mM Tris-HCl pH 7.4, 150 mM NaCl, 10 % Glycerol, 1 mM DTT). Native protein extracts of 50 OD cells (750 µl) expressing MBP-β7-CT or MBP control were incubated with the beads for 90 min at 4 °C, rotating. Afterwards, washing was performed 2x with 1 ml Buffer A and 3x with 1 ml Buffer B (Buffer A + 1 mg/ml BSA). Binding was proceeded with native protein extracts of 50 OD cells expressing different Ump1 variants (1-148Ump1 and 16-148Ump1) for 90 min at 4 °C, rotating. In the end, samples were washed 1x with 0.5 ml Buffer B and 2x with 0.5 ml Buffer A and eluted in 40 µl 1x Sample Buffer (5x Sample Buffer: 312,5 mM Tris pH 6.8, 10 % SDS, 50 % glycerol, 0.01 % bromophenol blue, 5 % β-mercaptoethanol) at 99 °C for 5 min. Samples were analyzed by 12 % SDS-PAGE and immunoblot using anti-MBP and anti-Ump1 antibodies.
